# Supplementary material for: A UMLS-based spell checker for natural language processing in vaccine safety
Source: BMC Med Inform Decis Mak. 2007 Feb 12;7:3. doi: 10.1186/1472-6947-7-3 (PMC1805499; doi:10.1186/1472-6947-7-3)
Supplement: Additional file 13 — RAP application directory. Sets up directory for RAP (RDF application for PHP) source code [file 1472-6947-7-3-S13.gz › rap/doc/tests/Test_Manipulate_DbModel.htm]

Test Set Database Connection


### RDF API for PHP V0.9.1

# Test: Manipulate DbModel

This example is part of the RAP - Rdf API for PHP documentation.

  

### 1. Connect to rdf database (e.g. Access via ODBC)

Set the system DSN for the particular database file (control pannel -> administrative
tools -> data sources (odbc) -> system DSN).

`// Include RAP  
define("RDFAPI_INCLUDE_DIR", "./../api/");  
include(RDFAPI_INCLUDE_DIR . "RDFAPI.php");  
   
// Connect to MsAccess database (rdf_db DSN)   
// using connection settings defined in constants.php :  
$rdf_database = ModelFactory::getDbStore();`

Read more about Setting Database Connection.

  

### 2. Load and show a DbModel

`// Load the model with modelURI "example1.rdf"  
$dbModel = $rdf_database->getModel("example1.rdf");   
  
// Output the model as HTML table  
$dbModel->writeAsHtmlTable();`

  

|  |  |  |
| --- | --- | --- |
| **Note:** |  | getModel() loads into memory only the modelURI and a database internal modelID, it also sets a database connection for the DbModel. This saves main memory and allows to gain advantage of database's indexing capabilities. |

**Output:**  
  

|  |  |  |  |
| --- | --- | --- | --- |
| **Base URI:** http://www.w3.org/Home/Lassila.rdf# | | | **Size:** 4 |
| **No.** | **Subject** | **Predicate** | **Object** |
| 1. | Resource: http://www.w3.org/Home/Lassila | Resource: http://description.org/schema/Creator | Blank Node: b85740 |
| 2. | Blank Node: b85740 | RDF Node: http://www.w3.org/1999/02/22-rdf-syntax-ns#type | Resource: http://description.org/schema/Person |
| 3. | Blank Node: b85740 | Resource: http://example.org/stuff/1.0/Name | Literal: Ora Lassila **(rdf:datatype="http://www.w3.org/TR/xmlschema-2#string")** |
| 4. | Blank Node: b85740 | Resource: http://example.org/stuff/1.0/Email | Literal: lassila@w3.org |

  
  

### 3. Add a statement to the DbModel

`// Ceate a new statement  
$statement = new Statement(new Resource("http://www.w3.org/Home/Lassila"),  
            
new Resource("http://description.org/schema/Description"),  
            
new Literal("Lassilas persönliche Homepage", "de"));  
  
// Add the statement to the DbModel  
$dbModel->add($statement);  
  
// Output the string serialization of the DbModel  
echo $dbModel->toStringIncludingTriples();`

**Output:**

MemModel[baseURI=http://www.w3.org/Home/Lassila.rdf#; size=5] Triple(Resource("http://www.w3.org/Home/Lassila"),
Resource("http://description.org/schema/Creator"), bNode("b85740"))
Triple(bNode("b85740"), Resource("http://www.w3.org/1999/02/22-rdf-syntax-ns#type"),
Resource("http://description.org/schema/Person")) Triple(bNode("b85740"),
Resource("http://example.org/stuff/1.0/Name"), Literal("Ora Lassila",
datatype="http://www.w3.org/TR/xmlschema-2#string")) Triple(bNode("b85740"),
Resource("http://example.org/stuff/1.0/Email"), Literal("lassila@w3.org"))
Triple(Resource("http://www.w3.org/Home/Lassila"), Resource("http://description.org/schema/Description"),
Literal("Lassilas persönliche Homepage", lang="de"))

  

### 4. Search statements

`// Search for statements having object $literal  
$literal = new Literal("Lassilas persönliche Homepage",
"de");  
$res = $dbModel->find(NULL, NULL, $literal);  
  
// Output the result  
$res->writeAsHtmlTable();`

**Output:**

|  |  |  |  |
| --- | --- | --- | --- |
| **Base URI:** http://www.w3.org/Home/Lassila.rdf# | | | **Size:** 1 |
| **No.** | **Subject** | **Predicate** | **Object** |
| 1. | Resource: http://www.w3.org/Home/Lassila | Resource: http://description.org/schema/Description | Literal: Lassilas persönliche Homepage **(xml:lang="de")** |

  
  

### 5. Replace nodes and serialize the DbModel to XML/RDF

`// replace a literal  
$dbModel->replace(NULL, NULL, new Literal("Lassilas pers�nliche Homepage", "de"),  
                             
new Literal ("Lassila's personal Homepage", "en"));  
  
// Serialize to RDF  
$dbModel->writeAsHtml();`

**Output:**

<?xml version='1.0' encoding='UTF-8'?>
